# Vitamin A Metabolism: An Update

**DOI:** 10.3390/nu3010063

**Published:** 2011-01-12

**Authors:** Diana N. D’Ambrosio, Robin D. Clugston, William S. Blaner

**Affiliations:** Department of Medicine and Institute of Human Nutrition, College of Physicians and Surgeons, Columbia University, New York, NY 10032, USA; Email: dd2244@columbia.edu (D.N.D.); rdc2132@columbia.edu (R.D.C.)

**Keywords:** chylomicron, carotenoid, retinol-binding protein (RBP), lecithin:retinol acyltransferase (LRAT), hepatocyte, hepatic stellate cell, adipocyte

## Abstract

Retinoids are required for maintaining many essential physiological processes in the body, including normal growth and development, normal vision, a healthy immune system, normal reproduction, and healthy skin and barrier functions. In excess of 500 genes are thought to be regulated by retinoic acid. 11-*cis*-retinal serves as the visual chromophore in vision. The body must acquire retinoid from the diet in order to maintain these essential physiological processes. Retinoid metabolism is complex and involves many different retinoid forms, including retinyl esters, retinol, retinal, retinoic acid and oxidized and conjugated metabolites of both retinol and retinoic acid. In addition, retinoid metabolism involves many carrier proteins and enzymes that are specific to retinoid metabolism, as well as other proteins which may be involved in mediating also triglyceride and/or cholesterol metabolism. This review will focus on recent advances for understanding retinoid metabolism that have taken place in the last ten to fifteen years.

## Abbreviations:

ABCA1: ATP-binding cassette, sub-family A, member 1; ABCR: ATP binding cassette transporter; APO: apolipoprotein; ARAT: acyl-CoA:retinol acyltransferase; ATGL: adipose triglyceride lipase; BCMO1: β-carotene-15,15′-monooxygenase; BCMO2: β-carotene-9′,10′-monooxygenase; CEL: carboxyl ester lipase; CRABP: cellular retinoic acid-binding protein; CRBPI, -II, and -III: cellular retinol-binding protein, type I, -type II and -type III; DGAT1: diacylglycerol acyltransferase 1; GI: gastrointestinal; GLUT4: glucose transporter 4; HDL: high density lipoprotein; HPSG: heparin sulfate proteoglycans; HSC: hepatic stellate cell; HSL: hormone sensitive lipase; ISX: intestine specific homeobox; LDL: low density lipoprotein; LPL: lipoprotein lipase; LRAT: lecithin:retinol acyltransferase; LRP: LDL receptor-related protein; MEF: myocyte enhancer factor; NAFLD: non-alcoholic fatty liver disease; PLRP1: pancreatic lipase related protein 1; PLRP2: pancreatic lipase related protein 2; PPAR: peroxisome proliferator-activated receptor; PPRE: peroxisome proliferator-activated receptor response elements; PTL: pancreatic triglyceride lipase; RBP: retinol-binding protein; RDH: retinol dehydrogenase; REH: retinyl ester hydrolase; RPE: retinal pigment epithelium protein; siRNA: small inhibitory RNA; SNP: single nucleotide polymorphism; SR-B1: scavenger receptor class B, type I; STRA6: stimulated by retinoic acid 6; TTR: transthyretin; VLDL: very low density lipoprotein; WT: wild type.

## 1. Introduction

It is nearly 100 years since the identification of vitamin A [1] in 1913 by McCollum and Davis [2], but much still remains to be learned about natural retinoid (vitamin A) metabolism and actions in the body. Many major questions regarding how retinoids are taken up from the diet and the molecular events important to retinoid storage and metabolism within specific cells and tissues need to be answered. Nevertheless, in the near 100 years that have gone by since the work of McCollum and Davis, a very extensive literature focused on retinoid metabolism and actions has accumulated. Our goal is to review important advances that have been made during the last 10 to 15 years toward understanding retinoid metabolism. This review will not concentrate on earlier work, as we will only briefly discuss what is known regarding retinoid metabolism from earlier published work to facilitate understanding of recent advances. For more information regarding seminal older research, the reader is referred to a number of extensive reviews published in the 1980s and 1990s to which this will serve as an update [3,4,5,6]. 

This review will focus primarily on (i) the intestinal absorption and metabolism of retinoid within the enterocyte; (ii) retinoid uptake, processing, and storage within the liver, where 70% of the retinoid stored within the body is located; and (iii) retinoid storage and metabolism in a number of extrahepatic tissues that have been the focus of recent research interest or where retinoid uptake and metabolism may be an integral component of the physiology of the tissue. The review will be primarily focused on mammals, with a few pertinent references to zebrafish, chickens and other non-mammalian species. We will not consider the molecular actions of retinoids in regulating retinoid-responsive transcription or their non-transcriptional actions. We will also not consider the metabolism of retinol to retinal or retinoic acid. Similarly, the potential roles of retinoids in either the prevention or causation of disease will be briefly discussed but not extensively covered. 

## 2. Metabolism within the Gastrointestinal (GI) Tract

**Figure 1:**
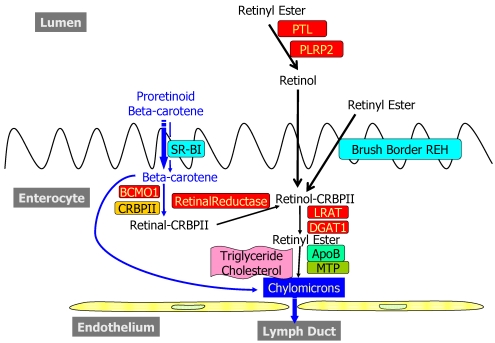
General scheme for the uptake and metabolism of dietary retinoids and proretinoid carotenoids within the intestine. Dietary proretinoid carotenoids, like β-carotene, are taken up into the enterocyte through a process that involves SR-B1. Once inside the enterocyte, β-carotene can be acted upon by BCMO1 and either converted to retinal, which binds CRBPII, or can be incorporated intact and unmodified along with dietary fat and cholesterol into nascent chylomicrons. The retinal produced from β-carotene cleavage must undergo reduction to retinol. This is catalyzed by one or more not well-characterized retinal reductases. Upon conversion to retinol, the retinol formed from dietary proretinoid carotenoids is metabolically indistinguishable from retinoid arriving in the diet as preformed retinoid. Dietary retinyl ester is either hydrolyzed in the lumen of the intestine by PTL or PLRP2 or undergoes hydrolysis at the intestinal brush border catalyzed by a brush border REH. Retinol taken into the enterocyte binds to CRBPII and is esterified to retinyl ester. In response to a physiological challenge of retinol, LRAT will catalyze approximately 90% of retinyl ester formation, while the intestinal acyl‑CoA:retinol acyltransferase, DGAT1, catalyzes the remainder of retinyl ester formation. The resulting retinyl ester is then packed along with dietary fat and cholesterol into nascent chylomicrons, which are secreted into the lymphatic system.

Retinoid metabolism within the GI tract occurs predominantly within the proximal portion of the small intestine and involves metabolic events that occur both in the lumen, as well as within the enterocyte [3,4,5,6]. These events are summarized in Figure 1 and are discussed below. For optimal retinoid absorption, fat must be consumed along with the newly ingested retinoid. This fat is needed to facilitate retinoid entry into enterocytes from the lumen of the gut. In addition, a fat load is needed to allow for optimal chylomicron formation, since retinoids, like other dietary lipids, enter the body as a component of nascent triglyceride-rich chylomicrons. 

The intestine is the primary tissue within the body where dietary proretinoid carotenoids, like β-carotene, are converted to retinoid. Dietary proretinoid carotenoids, as well as non-proretinoid carotenoids like lycopene and lutein, are incorporated into nascent chylomicrons and thus enter into the general circulation and the body.

### 2.1. Dietary Forms and Metabolism in the Lumen of the Intestine

Retinoid arrives from the diet either as preformed retinoid, consisting predominantly of retinol and retinyl ester, or as proretinoid carotenoids, which can be converted to retinoid within the intestine and other tissues. Human serum contains β-carotene, α-carotene, cryptoxanthin, lycopene, and lutein as major components, with smaller concentrations of zeaxanthin, other xanthophylls, and polyenes such as phytofluene and phytoene, which are all acquired from the diet [7].

The key digestive processes that occur within the lumen of the intestine include the physical release of dietary retinoids and proretinoid carotenoids from the food matrix and their emulsification with dietary fatty acids and bile acids. Emulsification with free fatty acids and bile salts is required to facilitate uptake of the highly insoluble retinoids and carotenoids into enterocytes from the lumen [3,4,5,6].

Dietary retinol is taken up directly from the lumen into the enterocyte; however, dietary retinyl esters must first undergo enzymatic hydrolysis within the lumen or at the enterocyte brush border to allow for uptake of the hydrolysis product retinol [3,4,5,6]. The identities of pancreatic enzymes that act in a physiologically significant manner in retinyl ester hydrolysis within the lumen were explored systematically in the last decade using both induced mutant mice and biochemical approaches [8,9]. Weng *et al.* reported studies of dietary cholesteryl ester and dietary retinyl ester absorption in wild type (WT) and carboxyl ester lipase (CEL) knockout mice [8]. These authors showed that, compared to WT mice, mice totally deficient in CEL absorbed only about 50% of the cholesterol provided as cholesteryl ester. Although earlier published work had proposed that CEL acted importantly within the lumen to catalyze retinyl ester hydrolysis, WT and CEL-deficient mice absorbed similar amounts of retinol when it was provided in a gavage as retinyl ester. Based on these findings, Weng *et al.* concluded that enzymes other than CEL must participate in the hydrolysis of dietary cholesteryl esters and retinyl esters within the GI tract [8]. This group of investigators subsequently reported studies that involved the separation and partial purification of pancreatic CEL and pancreatic triglyceride lipase (PTL) by DEAE-chromatography [9]. For both rats and mice, pancreatic retinyl ester hydrolase (REH) activity, measured by *in vitro* assay, was attributed mainly to PTL and, to a quantitatively lesser extent, to CEL. Purified human PTL was reported to exhibit similar enzymatic characteristics for both triglyceride hydrolysis and retinyl ester hydrolysis. Based on these biochemical data, it was concluded that PTL is the major pancreatic REH activity in rats and mice and is a catalytically active REH in humans, as well [9]. 

Purified horse PTL was reported by Reboul *et al.* to hydrolyze retinyl ester when provided either in triglyceride-rich lipid droplets, mixed micelles or vesicles [10]. It was further reported by these investigators that purified dog pancreatic lipase-related protein 2 (PLRP2), but not purified horse pancreatic lipase related protein 1 (PLRP1), catalyzes retinyl ester hydrolysis [10]. PLRP2-catalyzed retinyl ester hydrolysis required the presence of pancreatic colipase in order for activity to be observed. PLRP2 showed activity towards retinyl ester that had been incorporated into mixed micelles, but not emulsions. Based on these data, it was proposed that PTL and PLRP2 act synergistically within the lumen to catalyze dietary retinyl ester hydrolysis, enhancing the overall efficiency of retinoid absorption [10].

### 2.2. Metabolism and Processing within the Intestinal Mucosa

The intestine is the primary site of proretinoid carotenoid metabolism in the body. Dietary proretinoid carotenoids are taken up intact into the enterocyte, where they can undergo conversion to retinoid or be packaged unmodified into chylomicrons. During the past decade, considerable research activity has been focused on carotenoid uptake into, and conversion to retinoid, within the intestine. Similarly, there has been considerable progress made towards a better understanding of retinoid metabolism within the enterocyte.

#### 2.2.1. Uptake into and Efflux from the Enterocyte

Both *in vivo* studies, involving the use of mutant mouse models, and *in vitro* cell culture experiments have established scavenger receptor class B, type I (SR-B1) as a key a mediator for uptake of β-carotene from the intestinal lumen into the enterocyte [11,12,13]. Van Bennekum *et al.* studied cholesterol and β-carotene uptake by WT and SR-B1 knockout mice and concluded that SR-B1 is required for β-carotene absorption, at least for mice consuming a high fat diet [11]. These authors further showed that both SR-B1 and the plasma membrane fatty acid transporter CD36 can facilitate absorption of dietary cholesterol, but van Bennekum *et al.* were unable to establish whether SR-B1 acts essentially *in vivo* in facilitating this cholesterol uptake [11]. No evidence was obtained that CD36 acts in facilitating β-carotene absorption. SR-B1 expression in transfected COS-7 cells [11] and in intestinal Caco-2 cells [12] was found to confer on these cells the ability to take up β-carotene from mixed bile salt micelles, phospholipid small unilamellar vesicles, and triglyceride emulsions, thus, providing further evidence for a role for SR-B1 in β-carotene absorption. In addition to facilitating mucosal uptake of the proretinoid carotenoid β-carotene, SR-B1 also acts in facilitating uptake into the enterocyte of the non-proretinoid carotenoids lycopene and lutein [14,15].

Retinol uptake into cultured intestinal Caco-2 cells has been reported by During and Harrison to occur via both a saturable process, when retinol was provided at concentrations below 10 µM, and a nonsaturable process at higher retinol concentrations [12]. Expression of SR-B1 is not required for retinol uptake into Caco-2 cells, since knockdown of SR-B1 expression with small inhibitory RNAs (siRNAs) failed to influence retinol uptake by the cells [12]. Interestingly, inhibition of expression of the cholesterol efflux transporter ATP-binding cassette, sub-family A, member 1 (ABCA1) in Caco-2 cells through use of either siRNAs or the drug glyburide, which inhibits transport activity of ATP-binding cassette family members including ABCA1, diminished retinol efflux from the basolateral surface of the polarized cultures of Caco-2 cells [12]. These findings led to the proposal that retinol efflux from enterocytes is probably partially facilitated by the basolateral cholesterol transporter ABCA1. However, later studies by Reboul *et al.*, making use of both Caco-2 cells and ABCA1-deficient mice, failed to confirm this earlier observation [16]. Although Reboul *et al.* were able to convincingly demonstrate a role for ABCA1 in facilitating absorption of both α- and γ-tocopherol, their data provide no support for the idea that ABCA1 contributes to retinoid efflux, in nascent chylomicrons, from enterocytes [16]. 

#### 2.2.2. Enzymatic Conversion of Proretinoid Carotenoid to Retinoid

**Figure 2:**
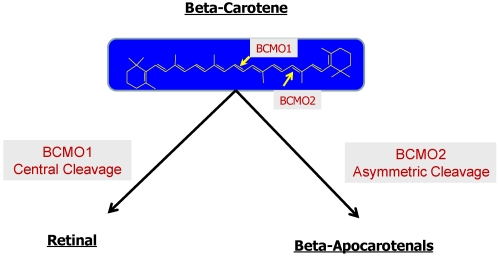
Carotenoids can undergo cleavage either symmetrically by BCMO1 or asymmetrically by BCMO2.

The enzymes involved in converting proretinoid carotenoids to retinoid were long a matter of heated research controversy [5,6]. From studies undertaken in the late 1950s and early 1960s, it was clear that enzymes existed within mammalian tissues that are able to cleave β-carotene, either symmetrically at its central 15,15′ carbon-carbon double bond, forming two molecules of retinaldehyde, or asymmetrically at other carbon-carbon double bonds, forming two products of unequal chain length (see Figure 2 below). Largely owing to technical difficulties in purifying and cloning these enzymes, from the 1970s through the 1990s, there was considerable debate as to whether only the central cleavage reaction contributed to retinoid formation or whether asymmetric cleavage also gave rise to quantitatively significant retinoid formation. This controversy was resolved in the last decade with the cloning and study of cDNA for two gene products, *Bcmo1* and *Bcmo2*, which encode enzymes able to catalyze central and asymmetric β-carotene cleavage, respectively. cDNAs for *Bcmo1* have now been cloned for the chicken [17], mouse [18,19,20], rat [21], and human [22,23], as well as for the fruit fly [24] and zebrafish [25]. Kiefer *et al.* reported cloning cDNAs for *Bcmo2* from the fruit fly, zebrafish, and the mouse and human [26]; whereas, Hu *et al.* reported cloning the ferret cDNA [27]. It is now established that only the *Bcmo1* gene product, which encodes an enzyme able to catalyze the central cleavage of β-carotene, is significant within the body for mediating retinoid formation from dietary proretinoid carotenoids [28,29].

Two structurally related proteins, β-carotene-15,15′-monooxygenase (BCMO1), encoded by *Bcmo1*, and β-carotene-9′,10′-monooxygenase (BCMO2), encoded by *Bcmo2*, are the sole mammalian enzymes known to cleave carotenoids. BCMO1 and BCMO2 are highly homologous to each other, with mouse BCMO1 and BCMO2 sharing 39% sequence identity. Each has a high degree of sequence homology to the retinal protein RPE65, which catalyzes the isomerization of all-*trans*-retinoid to the 11-*cis*-isomer for use in the visual cycle (see Section 5.3 below for more details). The older literature, based on the study of only partially purified enzyme, referred to BCMO1 as β-carotene-15,15′-dioxygenase, rather than as a monooxygenase [3,4,5,6]. However, a reevaluation of the reaction mechanism for this enzyme, employing recombinant protein, indicated that it acts through a monooxygenase, rather than a dioxygenase mechanism [30]. Biochemical data obtained by different laboratories regarding the properties and expression of mammalian BCMO1 are in general agreement [17,18,19,20,21,22,23]. BCMO1 is a soluble, Fe^2+^-containing, 63 kDa protein. It is expressed in the small intestine (at higher levels more proximal to the stomach), liver, kidney, lungs, skin, testis, the retinal pigment epithelium within the eye, and in a number of embryonic tissues. 

The biochemical properties and expression of BCMO2 have been less extensively studied than those of BCMO1. Like BCMO1, BCMO2 contains Fe^2+^ and has a cytoplasmic localization within the cell [26,27]. Both recombinant mouse and ferret BCMO2 catalyze cleavage of β-carotene at its 9′,10′ carbon-carbon double bond, and both will catalyze lycopene cleavage at its 9′,10′ carbon-carbon double bond [26,27]. In the mouse, *Bcmo2* is expressed in small intestine, liver, kidney, spleen, brain, and heart [26]. A similar tissue distribution has been reported for the ferret [27].

#### 2.2.3. The *Bcmo1* and *Bcmo2* Genes and Their Expression

The gene for *Bcmo1* and the regulation of its expression have been studied by a number of laboratories. The mouse and human genes for *Bcmo1* have both been shown to contain functional peroxisome proliferator-activated receptor (PPAR) response elements (PPREs) [31,32]. The PPRE present in the mouse gene is located within 60 bp upstream of the start site, and deletion or mutation of this element reduces promoter activity to its basal level [31]. Electrophoretic mobility shift assays established that PPARγ specifically binds this site, and administration of the PPARα/γ agonist WY14643 stimulated promoter activity in reporter assays, as well as increased BCMO1 protein expression in livers of mice administered the agonist. Using similar experimental approaches, Gong *et al.* established that the human *Bcmo1* gene contains a functional PPRE in its proximal promoter [32]. Moreover, these investigators demonstrated that the proximal promoter of the human *Bcmo1* gene also contains a functional myocyte enhancer factor 2 (MEF2) binding element and reported data suggesting that MEF2C, one member of the MEF2 transcription factor family, and PPARγ interact synergistically to transactivate *Bcmo1* expression. 

Other transcription factors may also play important roles in regulating *Bcmo1* expression, especially in intestine. Takitani *et al.* reported that intestinal *Bcmo1* mRNA expression was markedly increased in rats fed a retinoid-deficient diet and that expression was suppressed upon feeding of either all-*trans*- or 9-*cis*-retinoic acid [21]. However, although Takitani *et al.* demonstrated actions of retinoic acid in modulating intestinal *Bcmo1* expression, they couldn’t explain the molecular basis for their observation. Further insight was provided by subsequent work from Seino *et al.* [33] and Lobo *et al.* [13]. Seino *et al.* reported that the intestine-specific transcription factor Isx (intestine specific homeobox) plays a key role in regulating expression of *Bcmo1* in the mouse intestine [33]. These investigators, who generated and studied Isx-deficient mice through knock-in of LacZ, convincingly established that mRNA levels for both *Bcmo1* and *SR-B1* are greatly increased in the intestines of Isx-knockout mice. They further showed that severe vitamin A-deficiency markedly decreased *Isx* expression and that this was accompanied by an increase in *Bcmo1* expression in both duodenum and jejunum. Based on their data, Seino *et al.* suggested that *Isx* participates in the maintenance of retinoid metabolism by regulating *Bcmo1* expression in intestine. Lobo *et al.* carried this idea further by showing that retinoic acid, acting through retinoic acid receptors, induces *Isx* expression [13]. This effect of retinoic acid on *Isx* expression resulted in repression of both the *Bcmo1* and *SR-B1* genes. Through study of BCMO1-deficient mice, Lobo *et al.* were also able to establish that increased *SR-B1* expression and systemic β-carotene accumulation could be prevented through administration of dietary retinoid, which induced *Isx* expression, resulting in a downregulation of *SR-B1* expression and β-carotene uptake and systemic accumulation. Thus, the work of Lobo *et al.* established the existence of a diet-responsive regulatory network that controls β-carotene absorption and retinoid production through negative feedback regulation of *Isx* [13].

A number of single nucleotide polymorphisms (SNPs) in the human *Bcmo1* gene have been identified. One of these, a T170M missense mutation, results in a 90% reduction in enzyme activity when analyzed *in vitro* using purified recombinant enzymes [34]. A person identified as being heterozygous for the T170M mutation was reported in an earlier study to possess very high levels of serum β-carotene (14.8 µM), even though consuming only a typical Western diet not supplemented with β-carotene. Two common SNPs in the *Bcmo1* gene, R267S and A379V, alter β-carotene uptake in female volunteers receiving a pharmacologic dose (120 mg) of β-carotene [35]. Both variant alleles, when studied as recombinant proteins, showed reduced catalytic activity towards β-carotene. Carriers of both the 379V and 267S + 379V variant alleles displayed reduced ability to convert β-carotene, as indicated through reduced retinyl palmitate:β-carotene ratios in the triglyceride-rich lipoprotein fraction and increased fasting plasma β-carotene concentrations. Collectively, these data from SNP studies provide a compelling molecular explanation for why some individuals may be better able than others to take up and/or convert proretinoid carotenoids to retinoids. 

The structure and regulation of the *Bcmo2* gene have been much less extensively studied than is the case for *Bcmo1*. Mutations in the *Bcmo2* gene have been identified, and these influence carotenoid accumulation in chickens [36], cows [37] and sheep [38]. In domestic chickens, yellow skin coloration was found to be caused by a regulatory mutation that prevents expression of the *Bcmo2* gene in skin, allowing for accumulation of yellow carotenoids [36]. In cows, a mutation giving rise to a premature stop codon in the *Bcmo2* gene results in increased β-carotene concentrations in both serum and milk [37]. In sheep, a nonsense mutation in the *Bcmo2* gene is strongly associated with high levels of carotenoids deposited in fat, resulting in a yellow fat phenotype [38].

#### 2.2.4. Enterocyte Esterification of Retinol

Newly absorbed dietary retinol within the enterocyte must be esterified prior to its packaging as retinyl ester in nascent chylomicrons. The older literature had indicated that the intestine possesses two distinct enzyme activities able to synthesize retinyl esters from retinol [3,4,5,6]. One of these, lecithin:retinol acyltransferase (LRAT), catalyzes the transesterification of retinol employing a fatty acyl group present in the A1 position of a membrane phosphotidyl choline molecule. The other, acyl-CoA:retinol acyltransferase (ARAT), catalyzes the fatty acyl-CoA-dependent esterification of retinol. Studies by O’Byrne *et al.* of LRAT-deficient mice established, for mice receiving a physiologic dose of retinol (6 µg), that LRAT-catalyzed retinol esterification accounted for approximately 90% of intestinal retinyl ester formation [39]. Chylomicrons isolated from the dosed LRAT-deficient mice contained some retinyl ester, which was presumably synthesized by an intestinal ARAT, and relatively high levels of the free alcohol retinol, which were not observed in chylomicrons obtained from WT mice. Subsequent studies by Wongsiriroj *et al.* established that the enzyme diacylglycerol acyltransferase 1 (DGAT1), which catalyzes triglyceride synthesis from diacylglycerol and fatty acyl-CoA, acts as a physiologically significant ARAT in the mouse intestine [40]. Normally, for a physiological dose of retinol, DGAT1 accounts for approximately 10% of the retinol esterified in the intestine. However, the contribution that DGAT1 makes to intestinal retinol esterification becomes considerably greater upon administration of a large pharmacologic dose of retinol (1000 µg) [40]. Under physiologic conditions, LRAT and DGAT1 account for all retinol esterification within the enterocyte since no retinyl esters could be detected in chylomicrons isolated from LRAT/DGAT1-double knockout mice given a physiologic dose of retinol. However, when LRAT/DGAT1-double knockout mice were administered a pharmacologic dose of retinol, some retinyl ester could be detected in chylomicrons isolated from these mice, indicating the existence of other minor ARAT activities that can become active in response to excessive retinol intake [40]. In summary, LRAT accounts for the great majority of retinyl ester formed in the enterocyte upon consumption of normal dietary levels of retinoid; DGAT1, an intestinal ARAT, accounts for the remaining esterification activity. 

#### 2.2.5. Cellular Retinol-Binding Protein, Type II (CRBPII)

Retinoids are very insoluble in water and consequently within the aqueous environment of the body they are usually found bound to specific retinoid-binding proteins (see Table 1 below for a listing of retinoid-binding proteins). In the adult, CRBPII is reported to be expressed solely in the intestinal mucosa and is proposed to facilitate optimal retinol absorption from the diet [41]. Within the enterocyte, CRBPII represents 0.4–1.0% of the total cytosolic protein [42]. To investigate the physiological role of CRBPII, Li and colleagues generated and studied CRBPII-deficient mice [43]. When maintained on a retinoid-enriched diet, the knockout mice were found to have reduced (by 40%) hepatic retinoid stores, but the mutant mice grew and reproduced normally. However, when maternal dietary retinoid levels were reduced to marginal levels during the latter half of gestation, a 100% mortality rate, within 24 hours after birth, was observed for these litters [43]. These studies convincingly demonstrate that CRBPII acts to ensure adequate delivery of retinol to the developing fetus when dietary retinoid is limiting. Subsequent investigations making use of CRBPII-deficient mice bred into the LRAT-deficient background (lacking both *CrbpII* and *Lrat*) established that CRBPII metabolically channels retinol to LRAT for retinyl ester synthesis [40]. However, it could not be demonstrated experimentally that CRBPII directly prevents retinol from being acted upon *in vivo* by intestinal DGAT1 or other intestinal ARAT activities, as had been proposed in the older literature [4,5,6].

## 3. Chylomicrons and Their Metabolism in the Circulation

For uptake of dietary retinoid, retinyl ester is packaged along with other dietary lipids into nascent chylomicrons, which are secreted into the lymphatic system [3,4,5,6]. As mentioned above, dietary carotenoid that has not undergone conversion to retinoid is also incorporated into the nascent chylomicrons. After entering the general circulation, the nascent chylomicrons undergo a process of remodeling that involves primarily the hydrolysis of triglyceride by lipoprotein lipase (LpL) and the acquisition of apolipoprotein E (apoE) from the circulation, resulting in the formation of chylomicron remnants.

**Table 1:** Retinoid-binding proteins in the adult mouse ^a^.

Protein	Other Designations	Protein Family	Major Retinoid Ligands	Tissue Localization
RBP	RBP4	Lipocalin	all-*trans*-retinol	Many, with high levels in liver and adipose
IRBP	RBP3	−	all-*trans*-retinol	Retina
			11-*cis*-retinal	
CRBPI	RBP1	iLBP	all-*trans*-retinol	Many, with high levels in liver, kidney, testis, eye, lung
			all-*trans*-retinal	
CRBPII	RBP2	iLBP	all-*trans*-retinol	Small intestine
			all-*trans*-retinal	
CRBPIII	RBP7	iLBP	all-*trans*-retinol	Heart, muscle, adipose, mammary
CRABPI	RBP5	iLBP	all-*trans*-retinoic acid	Ubiquitous expression, with high levels in brain, skin and testes
CRABPII	RBP6 ^b^	iLBP	all-*trans*-retinoic acid	Primarily skin; also found in mammary, uterus, kidney, prostate and olfactory epithelium
CRALBP	RLBP1	CRAL_Trio	11-*cis*-retinal	RPE, retina, ciliary body, cornea, pineal gland, optic nerve, brain
			11-*cis*-retinol	
			9-*cis*-retinal	

^a^ The nomenclature for the retinoid-binding proteins in the literature is inconsistent between species. Thus, for clarity, this table contains alternative names for each protein in the adult mouse only. Alternative names may be different in other species, such as rat and human; ^b^ Only human CRABPII is designated RBP6; mouse CRABPII is not referred to as RBP6. Currently, there is no mouse form of RBP6.

It has long been established that 66–75% of dietary retinoid (chylomicron and chylomicron remnant retinoid) is taken up by the liver where it is stored in hepatic stellate cells (HSCs), with the remainder being cleared by peripheral tissues [44]. However, the significance of this early observation for understanding retinoid metabolism has been greatly underappreciated. Many general reviews and textbooks describe how tissues acquire needed retinoid as retinol bound to its specific binding protein, retinol-binding protein (RBP [45], also referred to as RBP4 in the literature). But these general texts often fail to consider the contribution that postprandial retinoids, present in chylomicrons and their remnants, make to tissue retinoid pools. Yet, 25–33% of all the dietary retinoid that is absorbed by the intestine is delivered via chylomicrons and their remnants to tissues other than the liver. The physiological importance of the postprandial retinoid delivery pathway is underscored by the general good health of humans who lack RBP [46], as well as RBP-deficient mice [47]. Both humans and mice that lack RBP are physiologically normal. Thus, if these humans or animal models are provided retinoid regularly, at normal levels in the diet, the postprandial retinoid delivery pathway is sufficient to meet tissue requirements for retinoid. It should be noted for completeness that the circulation also contains low levels of retinoic acid, which can contribute significantly to tissue retinoic acid pools [48].

The processes through which chylomicron/chylomicron remnant retinoid is absorbed by peripheral tissues are only now starting to be explored (see Section 5.2 below for more detail). LpL can hydrolyze retinyl ester present in chylomicrons, and it has been proposed that retinyl ester hydrolysis facilitates retinol uptake by peripheral tissues [49]. For many tissues, there is now growing evidence that LpL facilitates uptake of postprandial retinoid into tissues. Studies involving the use of different perturbations to LpL activity and/or levels within tissues have established that LpL acts to modulate postprandial retinoid uptake by skeletal muscle, heart and adipose tissue [49,50], mammary tissue and milk [51,52], and probably lung [53].

## 4. Hepatic Retinoid Metabolism

The liver is the major site of retinoid metabolism and storage in the body [3,4,5,6]. There are two hepatic cell types important to these processes: the parenchymal cells (also known as hepatocytes) and the stellate cells (also known as fat-storing cells, lipocytes, Ito cells, and perisinusoidal cells). The hepatocytes comprise approximately 66% of cells in the liver and contain 90% of the total protein mass [54,55,56]. The hepatic stellate cells (HSCs) are relatively much smaller and less abundant. The HSCs comprise only 6–8% of cells in the liver and contain 1% of hepatic protein [54,55,56]. It is well-established that hepatocytes are involved centrally in the uptake and processing of retinol in the liver, and that HSCs play a central role in hepatic retinoid storage. This section of the review will report on recent advances in our understanding of retinoid metabolism in hepatocytes and HSCs, including the uptake and processing of chylomicron retinyl ester by the hepatocyte, transfer of retinoid to the HSC, storage of retinoid in the HSC and hepatic retinol mobilization. 

### 4.1. Uptake and Processing of Chylomicron Retinyl Ester by the Hepatocyte

#### 4.1.1. Hepatic Chylomicron Remnant Receptors

When the retinyl ester-containing chylomicron remnant arrives at the liver, it passes into the space of Disse (located between the endothelium and the hepatocyte) in a process referred to as sieving [57]. Only remnants of appropriate size can pass through, while larger particles, including whole chylomicrons, are excluded. Once inside, the remnant is taken up exclusively by hepatocytes by one of two possible receptor-mediated pathways. The topic of receptor-mediated remnant uptake has been extensively and well reviewed by Cooper, and the reader is referred to this work for more detail [58]. Cooper recounts that one receptor-mediated pathway involves direct uptake by the low density lipoprotein (LDL) receptor, which has a high affinity for the apoE-rich chylomicron remnant particles, and internalization via endocytosis. If the LDL receptor is absent, down-regulated or saturated, the remnants may be sequestered in the space of Disse by binding to heparin sulfate proteoglycans (HSPGs), mediated by apoE. The remnants may also be sequestered through binding to hepatic lipase, which is enhanced by the presence of apoB. The remnants may eventually be transferred to LDL receptors as they become available or, if the remnants acquire enough apoE, transferred to an alternative receptor, the LDL receptor-related protein (LRP). LpL, acquired by the remnants during their formation, can facilitate uptake of remnants by LRP. It has not yet been established the extent to which each receptor contributes to chylomicron remnant-retinyl ester removal, but it likely depends on the metabolic state of the animal, which in turn can influence the amount of apoE being secreted. 

The importance of HSPGs in chylomicron remnant uptake was demonstrated by Zeng *et al.* who showed that remnant binding to the hepatocyte is dependent on both the expression of HSPG core proteins and the functionality of HSPG heparin sulfate chains [59]. Remnant binding in HepG2 cells was significantly decreased by antisense oligonucleotide knockdown of HSPG, antibodies to heparin sulfate, heparinase treatment and by various disruptions of the heparin sulfate chains, including inhibition of glycosylation. More recently, it’s been shown *in vivo* that the primary HSPG core protein involved in hepatic clearance of remnants is syndecan-1, which facilitates lipoprotein binding in the space of Disse [60]. 

Though less understood, evidence for a third hepatic remnant uptake pathway is emerging. Studies in normal and apoE-deficient mice fed low- or high-fat diets showed that plasma clearance of chylomicron remnants is delayed with non-lipolyzed particles and is inhibited by lactoferrin, which blocks LRP [61]. When lipolysis was restored with the addition of hepatic lipase, no difference in uptake was observed between the two diet groups or in the presence and absence of apoE. This provided evidence for an apoE-independent pathway that functions through LRP. This idea was further investigated by Out *et al.*, who proposed a mechanism involving interactions between remnant phospholipids and a receptor on the surface of hepatocytes, SR-BI [62]. In SR-BI-deficient mice, triglyceride-rich chylomicron-like particles associate significantly less with isolated hepatocytes compared to WT mice, with a concomitant delay in postprandial triglyceride clearance [62]. Adenovirus-mediated hepatic overexpression of *SR-BI* significantly decreased serum cholesterol, phospholipids and TG [63]. While a role for SR-BI in chylomicron remnant metabolism in the liver is likely, it remains to be determined if and how it may interact with the other receptor systems. 

#### 4.1.2. Retinyl Ester Hydrolysis in the Hepatocyte

Upon entry into the hepatocyte, the retinyl ester is associated with early endosomes and undergoes rapid hydrolysis [64]. The hydrolysis of retinyl ester is carried out by a number of enzymes referred to in the literature as REHs, carboxylesterases and/or lipases. 

The first well-characterized REH was the bile salt-dependent CEL (see above for more details regarding CEL actions in the intestine), which is a potent bile salt-dependent REH *in vitro* [65,66]. Hepatic CEL was found to have very close similarity to pancreatic CEL in terms of mRNA sequence, enzymatic activity and antibody recognition [67]. Like pancreatic CEL, hepatic CEL is also a secreted enzyme, so it was first hypothesized to be involved in retinyl ester hydrolysis in the space of Disse [68]. However, it was shown that lack of *CEL* expression does not affect uptake of dietary chylomicron remnant-retinyl ester in the liver, and furthermore, the livers of CEL-deficient mice display similar REH activity compared to wild type livers [69]. The presence of REH activity in the liver distinct from CEL was confirmed by the discovery of neutral, bile acid-independent REH activity in rat liver homogenates that localized to the microsomal fraction, consistent with a role in retinyl ester hydrolysis in the plasma membrane and/or endosome, and was not cross-reactive with antibodies to the pancreatic enzyme [70]. It was later found that this activity was stimulated by the presence of apo-CRBPI at physiological concentrations, suggesting apo-CRBPI may be a regulator of retinyl ester hydrolysis *in vivo* [71]. Acidic bile salt-independent REH activity has also been observed in rat liver plasma membrane and endosomal fractions [72]. It has been postulated that the neutral REH acts on chylomicron remnant-retinyl ester at the cell surface and when it enters the early endosome, and as the pH drops in the late endosome, retinyl ester hydrolysis is continued by the acidic REH [73]. 

Another group of enzymes proposed to be important to retinyl ester hydrolysis in the liver is the carboxylesterases. Although over 30 liver carboxylesterases with various oxyester substrates have been identified, most of these enzyme activities are thought to arise as the gene products of five major loci in linkage group V and are referred to as ES-2, ES-3, ES-4, ES-10 and ES-15 [74]. Of these five enzymes, ES-2, ES-4 and ES-10 have been shown specifically to possess REH activity in the liver *in vitro* [75,76]. ES-4 functions primarily as a thioesterase to catalyze the hydrolysis of long-chain acyl Co-A [75], but it has also been shown to hydrolyze retinyl palmitate [77]. ES-2 and ES-10 have been shown to function as neutral bile salt-independent REHs. Sun *et al.* purified REH activity from rat liver microsomal fractions that reacted with antibodies against ES-2, showed substrate preference for retinyl palmitate and had a pH optimum of 7 [76]. Similarly, they were able to purify REH activity that corresponded to the protein size and amino acid sequence of ES-10 and also demonstrated immunoreactivity to antibodies directed against ES-10. More recently, ES-22 has been reported to be a hepatic REH. Schreiber *et al.* identified ES-22 as a hepatocyte-expressed esterase that localizes to the ER [78]. These investigators showed that ES-22 specifically hydrolyzes retinyl palmitate, but not triolein or cholesteryl oleate. Additionally, overexpression of ES-22 inhibited the accumulation of retinyl esters in COS-7 cells. 

A number of *in vitro* studies have also identified some well characterized lipases that possess REH activity, including hepatic lipase [79], LpL [49,50], PTL [9,10], and hormone sensitive lipase (HSL) [80]. Mello *et al.* have shown that, of these, only hepatic lipase and LpL are expressed in the liver (mRNA levels of PTL and HSL in all liver cell types were very low) [81]. Hepatic lipase is exclusively expressed in hepatocytes and is the only secreted lipase expressed in these cells [81]. It is secreted by the hepatocyte into the space of Disse, where it can function in the binding and uptake of chylomicron remnants and possibly in the hydrolysis of chylomicron remnant retinyl ester, though there is currently no direct evidence for the latter possibility. LpL is expressed at very low levels in hepatocytes and HSCs, but its expression is induced 32-fold in activated HSCs [81]. Thus, Mello *et al.* propose that it is unlikely LpL functions in the hydrolysis of newly absorbed hepatic chylomicron remnant-retinyl ester, but rather may have a role in the hydrolysis of lipid droplet retinyl esters in activated HSCs [81]. How this might occur is unclear, given that LpL is a secreted enzyme, and there is no evidence for it acting intracellularly. A summary of proposed REHs in hepatocytes can be found in Table 2. 

The physiological significance of the many lipid hydrolases and carboxylesterases in the liver remains to be indentified. However, this uncertainty is true for many other tissues in the body as well; *in vivo* retinyl ester hydrolase activity has only been determined for a number of these enzymes. For example, it is clear that PTL and PLRP2 are physiologically significant retinyl ester hydrolases in the intestine. As discussed in further detail below, LpL-catalyzed retinyl ester hydrolysis is important for facilitating postprandial retinol uptake into adipose tissue, heart and skeletal muscle. And similarly, it is known that HSL catalyzes retinyl ester hydrolysis in adipocytes to allow for retinol mobilization into the circulation. With these exceptions, the identities of most other physiologically significant retinyl ester hydrolases remain obscure. It is likely that several other enzymes will be shown to have physiologically signifcant and distinct roles in catalyzing this reaction. With respect to the liver, we speculate that this may include hepatic lipase in hepatocytes and HSL in HSCs. Much work remains to elucidate the roles of these many enzymes in the various tissues that function in the uptake and metabolism of retinoid in the body.

**Table 2:** Proposed Hepatic Retinyl Ester Hydrolases (REHs).

Protein	Hepatic Cell Type
Bile salt-dependent REH (identified to be CEL)	Hepatocyte
Neutral, Bile salt-independent REH	Hepatocyte
Acidic, Bile salt-independent REH	Hepatocyte
ES-2	Hepatocyte
ES-4	Hepatocyte > HSC
ES-10	Hepatocyte > HSC
ES-22	Hepatocyte
Hepatic Lipase	Hepatocyte
LpL	HSC (activated)
ATGL	HSC

### 4.2. Transfer of Retinol from Hepatocytes to HSCs

After the chylomicron retinyl ester is hydrolyzed to retinol within the hepatocyte, it is then transferred to the HSC where it is re-esterified and stored in lipid droplets. This transfer occurs primarily in times of sufficient or excess retinoid intake, so that these retinoid stores may be called upon and mobilized in times of vitamin A deficiency. The mechanism of transfer has not been the focus of more recent research and thus, is not fully understood. A couple of possibilities exist and are discussed below. 

#### 4.2.1. RBP-Mediated Transfer

Early studies suggested that this transfer is mediated by retinol-binding protein (RBP). Blomhoff *et al.* reported that antibodies against RBP completely blocked the transfer of retinol from hepatocytes to HSCs [82]. Following loading of hepatocytes *in vivo* by intravenous injection of [^3^H]retinyl ester-labeled chylomicrons, livers were perfused *in situ* with either a control buffer or a buffer containing an excess of antibodies against RBP. In both groups, the radioactivity recovered 10 min after loading was significantly higher in hepatocytes than in HSCs; after 40 min of perfusion, the control group showed significantly higher ^3^H-cpms associated with the HSCs, while the HSCs from the livers perfused with the RBP antibodies still had less ^3^H-cpms than hepatocytes. A similar study by Senoo *et al.* showed that antibodies to RBP blocked its transfer from HepG2 cells to rat HSCs, when the cells were co-cultured [83]. This study also showed that RBP could be internalized by cultured HSCs when the cells were incubated with human RBP. RBP was first observed to be associated with vesicles close to the cell surface and membrane and was later found deeper in the cytoplasm within endosomes. The findings that RBP is able to be bound and internalized by HSCs and that antibodies to RBP inhibited transfer of retinol into HSCs was taken to suggest that RBP mediates this transfer. 

However, the generation of RBP-deficient mice allowed this hypothesis to be tested directly, and the results are not in agreement with these earlier proposals. Quadro *et al.* showed that, in 3- and 13‑week-old mice, there is no statistical difference in hepatic total retinol levels between WT and RBP-deficient mice; thus, the absence of RBP does not impair hepatic uptake of retinol [47]. It was later demonstrated that there is no quantitative or qualitative differences in the lipid droplets present in liver sections from RBP-deficient compared to WT mice, suggesting that retinol transfer to HSCs for storage is unaffected by the absence of RBP [84]. These studies provide strong evidence that RBP does not act in an essential manner in the transfer of retinol from hepatocytes to HSCs. 

#### 4.2.2. CRBPI-Mediated Transfer

The involvement of CRBPI in the transfer process has been suggested by data from Ghyselinck *et al.* [85], whose studies of CRBPI-deficient mice suggest that CRBPI may mediate the transfer of retinol from hepatocytes to HSCs [85]. CRBPI-deficiency was found to result in significantly lower hepatic retinyl ester levels, with mutant mice having a 50% reduction in levels of retinyl palmitate, the main retinyl ester form found in the liver. Light microscopy also revealed that the HSCs of CRBPI-deficient mice are characterized by a reduction in both the number and size of lipid droplets. This suggests a decrease in retinyl ester synthesis that likely occurs due to impaired delivery of retinol to the retinyl ester-synthesizing enzyme LRAT, which is highly expressed in HSCs [86]. These studies clearly indicate that CRBPI is necessary for assuring efficient retinol esterification and storage *in vivo*. Future studies are needed to confirm how the presence and absence of CRBPI affects total retinol levels specifically in hepatocytes and HSCs. 

### 4.3. Storage of Retinoid in the HSC as Retinyl Ester in Lipid Droplets

#### 4.3.1. The role of HSC Lipid Droplets in Retinoid Storage

The most distinguishing feature of the HSC is the presence of numerous retinyl ester-containing lipid droplets in the cytoplasm, which have recently been proposed to be specialized organelles for retinoid storage [86]. This idea arises from numerous studies showing the unique retinoid content of these droplets, their responsiveness to dietary retinoid status, their dependence on the synthesis of retinyl ester, and their loss in different types of hepatic disease. Taken together, these studies have demonstrated a strong regulatory role of retinoids in HSC lipid droplet physiology. This will be discussed below in more detail.

#### 4.3.2. Esterification of Retinol in the HSC

After transfer from the hepatocyte to the HSC, retinol is esterified back to retinyl ester for storage in HSC lipid droplets. Once in the HSC, retinol is bound by CRBPI and transferred to LRAT for esterification [87]. The binding of retinol to CRBPI is necessary for its transport in the aqueous environment of the cytosol and has been proposed to prevent acyl CoA-dependent enzymes in the liver from catalyzing retinyl ester formation [87]. Both LRAT and CRBPI are enriched in HSCs [5,6,86], and both proteins are needed to assure optimal HSC accumulation of retinoid stores. 

LRAT is the only known enzyme capable of esterifying retinol in the liver *in vivo*. Earlier *in vitro* studies suggested that an unidentified ARAT(s) may work in conjunction with LRAT to catalyze retinyl ester formation [88]. As mentioned above, DGAT1 has been shown to participate in retinol esterification *in vitro* [39,89,90] and in the intestine and skin *in vivo* [40,91]. Thus, DGAT1 became a candidate for *in vivo* esterification of retinol in the liver, as well. However, the generation and study of LRAT-deficient mice fail to support both possibilities. O’Byrne *et al*. show that the livers of LRAT-deficient mice have undetectable levels of retinyl ester and are completely void of lipid droplets [39]. These studies not only proved that LRAT is the only retinol esterifying enzyme present in the liver, but also that LRAT and/or its product retinyl ester is necessary for HSC lipid droplet formation. Around the same time, Liu and Gudas reported that disruption of the *Lrat* gene makes mice more susceptible to retinoid-deficiency [92]. After maintenance on a retinoid-deficient diet for six weeks, LRAT-deficient mice had significantly lower serum retinol levels than WT mice and undetectable levels of retinol in a number of tissues studied, including the liver. Thus, Liu and Gudas proposed that LRAT-deficient mice may serve as a readily inducible, and hence useful, model to study retinoid-deficiency [92].

#### 4.3.3. HSC Lipid Droplet Content and Effects of Dietary Retinoid Status

Moriwaki *et al.* reported the lipid composition of lipid droplets isolated from primary rat HSCs [93]. In rats maintained on a control diet, the mean percent lipid composition consisted approximately of 40% retinoid lipid and 60% non-retinoid lipid, broken down as follows: 39.5% retinyl ester, 31.7% triglyceride, 15.4% cholesteryl ester, 6.3% phospholipid, 4.7% cholesterol and 2.4% free fatty acids. The retinyl esters present in these droplets possess only long chain fatty acyl moieties, with retinyl palmitate followed in abundance by retinyl stearate, retinyl oleate and retinyl linoleate [94]. In the same study, the rats were maintained on four additional experimental diets containing either low or high retinol and low or high triglyceride. The investigators found that HSC lipid droplet retinyl esters, as well as other lipids, are decreased in response to a low retinol diet, and both retinoid and non‑retinoid lipids are elevated in response to a high retinol diet; however, neither retinoid nor non‑retinoid content is affected by low or high fat diets. This data was taken to indicate that the lipid composition of HSC lipid droplets is strongly influenced by dietary retinoid status, but not by dietary triglyceride intake.

#### 4.3.4. HSC Lipid Droplets in Hepatic Disease

Following acute liver injury, a wound healing response is initiated, which ultimately returns the liver to its healthy state [54,55,56]. However, when the liver is confronted with chronic injury, the result is often hepatic fibrosis, which is the formation of excess fibrous connective tissue as a reparative process to contain the site of injury. The most common causes of hepatic fibrosis include chronic hepatitis B and C infection, alcoholism, non-alcoholic fatty liver disease (NAFLD), and bile duct obstruction. Further progression of disease will lead to cirrhosis and eventually hepatocellular carcinoma. Seminal work by Leo and Lieber established that with this progression of liver disease in human subjects is the progressive depletion of hepatic retinoid [95]. These investigators found that there is a near 5-fold decrease in total hepatic retinol levels with the development of alcoholic hepatitis and another approximate 4-fold decrease with the development of cirrhosis. It remains unclear whether this loss of hepatic retinoid content is a causative agent or simply a consequence of disease.

As a result of injury, HSCs undergo a process of activation in which they transition from a quiescent state to a myofibroblastic phenotype [96,97]. Activated HSCs are characterized by increased synthesis of extracellular matrix and thereby become fibrogenic and proliferative [96,97]. There are currently several *in vivo* models of HSC activation, including carbon tetrachloride injection and bile duct ligation, and one commonly studied *in vitro* model, the culture of primary HSCs on plastic. It has been demonstrated that the changes in gene expression that accompany HSC activation and the loss of HSC retinyl ester lipid droplets are regulated differently in the *in vitro* model and the *in vivo* models [98]. Thus, the study of HSC activation can only be studied in the context of the disease model employed. 

Two hypotheses have been proposed to account for the loss of retinyl ester lipid droplets in hepatic disease. One theory is that HSC activation results in a “toxic burst” of transcriptionally active retinoids from the lipid droplet stores and, acting through the retinoic acid receptors and the retinoid X receptors (all of which are expressed in HSCs), lead to the altered gene expression patterns seen with activation [99,100]. The other proposes that the retinoid stores in these lipid droplets play a protective role, such that their presence buffers against hepatic insult [101]. When these stores are absent, an injury to the liver will result in fibrosis and hepatic disease. This second hypothesis seems unlikely considering LRAT-deficient mice are not predisposed to developing spontaneous hepatic fibrosis [39]. However, more definitive studies need to be conducted before the linkage between the loss of HSC retinoid stores and hepatic disease can be determined.

### 4.4. Mobilization of Retinol from Hepatic Stores to Peripheral Tissues

#### 4.4.1. Hydrolysis of HSC Lipid Droplet Retinyl Ester

As mentioned above, HSC lipid droplet retinyl ester stores must be mobilized in times of dietary retinoid-insufficiency to supply peripheral tissues with retinoid needed for maintaining various essential biological functions. Mobilization requires that the retinyl ester first be hydrolyzed back to retinol. It is currently not known which lipases are involved in the hydrolysis of HSC retinyl ester, but there are several candidate enzymes based on their REH activity and/or expression in HSCs (see Table 2). As discussed above, ES-2, ES-4 and ES-10 are three hepatic carboxylesterases shown to have REH activity [75,76]. Mello *et al.* show that ES-4 and ES-10 are expressed in HSCs, albeit at very low levels compared to hepatocytes [81]. Their REH activity and expression in HSCs make ES-4 and ES-10 strong candidates for HSC lipid droplet retinyl ester hydrolases. Another candidate for HSC retinyl ester hydrolysis is adipose triglyceride lipase (ATGL). ATGL is the key enzyme for triglyceride hydrolysis in adipocytes [102], and its lipase activity *in vivo* is dependent on coactivation by CGI‑58 [103]. Earlier studies suggested ATGL does not have REH activity, but these studies were done in the absence of CGI-58 [102]. Mello *et al.* found that ATGL is expressed highly in HSCs and propose that, in the presence of CGI-58, will have REH activity [81]. However, this possibility has not yet been demonstrated directly. 

#### 4.4.2. Role of RBP in Hepatic Mobilization of Retinol

Upon hydrolysis of HSC lipid droplet retinyl ester, retinol is thought to be transferred back to the hepatocyte, where it is bound by its specific transport protein, RBP. RBP is a 21 kDa protein with a single binding site for one molecule of all-*trans*-retinol, and the hepatocyte is the major (though not exclusive) site of RBP synthesis in the body [104]. Once bound by RBP, the retinol-RBP complex enters the bloodstream for transport to peripheral tissues. Secretion of RBP by the hepatocyte is highly regulated by the retinoid status of the animal, such that RBP secretion is blocked in times of dietary deficiency and restored upon retinol-repletion [105]. 

The importance of RBP in maintaining retinoid homeostasis in the body was demonstrated by Quadro *et al.* through generation of RBP-deficient mice [47]. These investigators show that, in 3- and 13-week-old mice, there is no statistical difference in hepatic total retinol levels between WT and RBP-deficient mice; thus, the absence of RBP does not impair hepatic uptake and accumulation of retinol. However, when these investigators measured hepatic and serum retinol levels in 5-month-old mice, they found that hepatic retinol and retinyl ester levels were significantly higher in RBP-deficient compared to WT mice and, additionally, serum total retinol levels were significantly lower. These data indicate that RBP-deficient mice can acquire hepatic retinol stores, but these cannot be mobilized. It was later shown using transgenic mice that express human RBP that extrahepatically synthesized RBP cannot be taken up by hepatocytes and cannot function in the mobilization of hepatic retinol stores [84]. Only RBP synthesized in the liver can mobilize hepatic retinol stores.

In the blood, RBP is found in a 1:1 protein-protein complex with a 55 kDa serum protein, transthyretin (TTR) [104]. The retinol-RBP-TTR complex is the predominant transport molecule through which retinol is delivered to peripheral tissues in the fasting circulation. In the absence of TTR, plasma retinol and RBP levels are only approximately 5% of those that are observed in matched WT mice [104]. It has been established that the association of RBP with TTR prevents filtration of the relatively small RBP molecule through the kidney glomeruli [106]. Studies by van Bennekum *et al.* employing TTR-deficient mice have demonstrated the importance of TTR in maintaining normal levels of retinol and RBP in the circulating plasma. Subsequent studies established that, when a physiologic dose of human retinol-RBP was injected intravenously into TTR-deficient mice, it was cleared more rapidly from the plasma and accumulated more rapidly in the kidneys of TTR-deficient than WT mice [106]. These data were taken by the authors to indicate that the reduced levels of retinol and RBP in the circulations of TTR-deficient mice arise, at least in part, due to increased filtration of the retinol-RBP complex. Other studies of TTR-deficient mice showed that the livers of these mutants have similar levels of retinol and retinyl ester compared to WT mice, but 60% higher levels of RBP protein [107]. These data indicated that TTR does not have a role in hepatic uptake or storage of dietary retinol, but suggested it may play a role in hepatic secretion of RBP. However, van Bennekum *et al.* were unable to establish directly that the absence of TTR affects RBP secretion from hepatocytes [106]. These investigators showed that cultured primary hepatocytes isolated from TTR-deficient mice accumulated RBP in their culture media to the same degree as hepatocytes from WT mice; thus, RBP was being secreted from hepatocytes at the same rate in the presence and absence of TTR. 

## 5. Uptake of Retinoids by Extrahepatic Tissues

**Figure 3:**
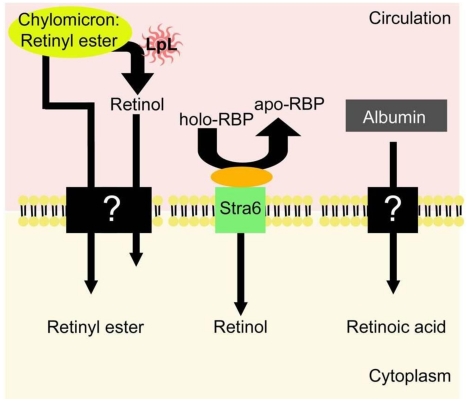
Uptake of retinoids into extrahepatic tissues. Retinoids in the circulation are present in several forms, including retinol bound to RBP (holo-RBP), retinyl esters in lipoproteins (primarily chylomicrons, but also VLDL, LDL, and HDL), and retinoic acid bound to albumin. The mechanisms that mediate cellular uptake of retinyl ester and retinoic acid are not fully understood. However, it has been established for certain tissues that LpL can hydrolyse retinyl ester to retinol, which can then be taken up by tissues and cells. The transmembrane protein STRA6 is able to bind holo-RBP and facilitate its uptake into cells.

In the fasting state, >95% of retinoid in the circulation is found as retinol bound to RBP (*i.e.*, as retinol-RBP). The remainder of circulating retinoid in the fasting circulation is comprised of a variety of low abundance species, including retinyl esters associated with lipoproteins (very low density lipoprotein (VLDL), low density lipoprotein (LDL), and high density lipoprotein (HDL)) and retinoic acid bound to albumin. In the postprandial state, chylomicron retinyl ester is elevated and can quantitatively predominate over retinol-RBP [6]. In this section, recent advances in our understanding of how retinol-RBP is taken up in extra-hepatic tissues will be discussed, as well as insights into mechanisms associated with the uptake of retinol from postprandial, chylomicron-remnant derived, retinyl ester (as summarized in Figure 3 below).

### 5.1. Uptake of Retinol-RBP from the Blood by Extrahepatic Tissues

While the spontaneous transfer of free retinol across the phospholipid bilayer is a possibility supported by experimental evidence, the existence of a cell-surface receptor for RBP has been postulated since the mid-1970s (as extensively summarized in [6]). In 2007, Kawaguchi *et al*. identified and studied a transmembrane-spanning protein STRA6 (stimulated by retinoic acid 6) that acts as a receptor for RBP in many, but not all, tissues (since it is not expressed in all retinoid metabolizing tissues) [108]. These authors demonstrated that (i) RBP can bind to STRA6 with high affinity, (ii) STRA6-transfected cells efficiently take up retinol, especially if LRAT is expressed within the cells, (iii) RNAi knockdown of STRA6 suppresses retinol uptake, and (iv) STRA6 is expressed in tissues consistent with its function as an RBP receptor. These experiments provide strong evidence that STRA6 can function as an RBP receptor and mediate the cellular uptake of retinol. Interestingly, a synergy exists between STRA6 and LRAT expression, such that cells expressing both proteins uptake relatively more retinol than cells expressing either protein individually [108,109]. This phenomenon indicates that conversion of retinol into retinyl ester by LRAT within the cell maintains the driving force for STRA6-mediated retinol uptake. This suggests a model whereby circulating retinol-RBP binds to STRA6 located on the cell surface, facilitating uptake of retinol and its conversion to retinyl ester by LRAT. 

The concept of STRA6-mediated cellular uptake of retinol has been extended to include STRA6-mediated efflux of retinol from the cell by von Lintig and colleagues. Cell culture experiments have shown that STRA6-expressing cells, preloaded with retinol, release more retinol into the culture medium than cells which do not express STRA6, a process which is RBP-dependent [109]. *In vivo* evidence of STRA6-mediated retinol efflux comes from observations made in the developing mouse embryo [110]. Embryonic STRA6 expression is upregulated in response to maternal dietary retinoid-excess. Similarly, STRA6 levels were elevated in LRAT-deficient mouse embryos, where intracellular retinol levels would be higher because of the incapacity to convert retinol to retinyl ester. These data are interpreted to indicate that STRA6 is upregulated to facilitate export of excessive levels of retinol from the cell [110]. These findings suggest that STRA6 acts as a bidirectional transporter of retinol, with intracellular retinol concentrations determining the polarity of transport.

The notion that STRA6 acts as a bidirectional retinol transporter is attractive as it resolves a theoretical paradox with regard to STRA6 expression [111]. If one accepts that STRA6 facilitates retinol uptake and that its expression is stimulated by retinoic acid, then in times of excess retinoic acid, STRA6 expression would increase and drive more potentially toxic retinoid into the cell. This detrimental scenario is avoided if we accept that STRA6 also facilitates retinol export. Here, excess retinoic acid and the associated increase in STRA6 would channel retinol out of the cell, thereby protecting it. It is possible that STRA6 functions at a basal level to import retinol into the cell to maintain intracellular retinoid homeostasis, and in times of retinol excess within the cell it can be upregulated and function in retinol export, protecting the cell from a potentially cytotoxic build up of retinoid.

The hypothesis that STRA6 expression is driven by retinoic acid is supported by studies in several cell lines [112,113,114,115]. But data from *in vivo* measurements of STRA6 expression are not in good agreement. High maternal dietary retinoid intake is associated with increased embryonic STRA6 expression [110], and retinoic acid has been shown to increase STRA6 expression in the lungs of neonatal rats [116]. However, these data conflict with observations from chick embryos, in which retinoic acid soaked beads had no effect on STRA6 expression [117]. If we consider the opposite extreme of retinoid nutriture, the data are also contradictory. Mouse embryos from dams deprived of dietary retinoid showed no change in STRA6 expression [110], whereas retinoid-deficient quail embryos upregulate STRA6 [117]. It is likely that control of STRA6 expression is tightly regulated at both the gene and protein levels, and depends on the cellular context and retinoid status of the animal. 

Irrespective of debate regarding the physiological function of STRA6, a role in cellular retinoid homeostasis is pointed to by the severe congenital abnormalities associated with STRA6 mutations in humans. Postulated loss-of-function mutations in STRA6 have been found in more than 20 individuals with syndromic anophthalmia/microphthalmia, in association with variable malformations of the heart, lungs, and diaphragm (Microphthalmic syndrome 9, OMIM 601186) [118,119,120,121,122,123,124,125]. Significantly, retinoid signaling is known to be important in the development of the eye [126,127], lungs [128,129], heart [130,131], and diaphragm [132,133]. Further, malformations in these tissues are commonly found as part of the maternal retinoid-deficiency syndrome [134]. The parallel between the effects of maternal retinoid-deficiency and STRA6 mutation supports the concept that STRA6 is needed for maintaining cellular retinoid homeostasis during organogenesis. Interestingly, there is a discrepancy between the severe outcome of STRA6 mutation in humans, and mutations of RBP, which yield only a mild clinical phenotype [46,47], leading some authors to speculate that STRA6 may have other unknown functions in addition to it being an RBP receptor [111]. 

### 5.2. Extrahepatic Uptake of Chylomicron Retinyl Ester

As mentioned above, nascent chylomicrons from the intestine are rapidly metabolized in the general circulation, yielding smaller lipoprotein particles called chylomicron remnants. It has been well established that while ~75% of postprandial retinyl ester is taken up by the liver, the remaining ~25% is taken up by extra-hepatic tissues, including white adipose tissue, skeletal muscle, heart, lungs, and kidneys [44,50,69]. The physiological importance of postprandial retinyl ester uptake into extra‑hepatic tissues was initially unclear; however, a growing body of evidence suggests that this source can be an important factor in maintaining retinoid homeostasis in specific tissues.

A physiologic role for uptake of postprandial retinyl ester was highlighted in mice lacking RBP [47]. Despite having only trace levels of circulating retinol and having an impaired ability to mobilize hepatic retinol, RBP-deficient mice are viable and fertile. These mice do have a transient visual defect, though this resolves with age (a further expansion of the RBP-deficient phenotype is provided below). The relatively benign phenotype of these mice led the authors to hypothesize that retinoid homeostasis was being maintained by uptake of postprandial retinoid. Investigation of RBP-deficient mice revealed that the mutant mice have a relatively high concentration of circulating retinyl ester in the chylomicron/VLDL plasma fraction, supporting the hypothesis that chylomicron-derived retinyl esters were important in maintaining their good health [135]. It was also found that the eye is particularly poor at taking up chylomicron-derived retinyl ester; this suggests why vision was impaired in these animals, whereas other organ systems were unaffected because of their ability to readily uptake postprandial retinyl ester [136]. Study of RBP-deficient mice also indicated that these mice can use postprandial retinyl ester to support normal embryogenesis [135]. This concept was investigated in more depth showing that retinyl ester is important for the establishment of fetal retinoid stores, whereas retinol-RBP has a more direct role in embryogenesis and organogenesis [137]. 

The contribution of postprandial retinyl ester uptake has also been studied in other tissues. In the lactating mammary gland, a contribution of retinyl ester into milk retinoid has been shown in non‑human primates and rodents [51,138,139,140]. The magnitude of this contribution is thought to reflect dietary retinoid intake, such that with increasing levels of retinoid consumed in the diet, the proportion of chylomicron-derived retinyl ester in the milk relative to retinol-RBP, also increases. In the extreme, it has been shown that chylomicron retinyl ester uptake can completely compensate for RBP-deficiency in mammary tissue, such that the retinoid content of milk in RBP-deficient mice is identical to age- and diet-matched WT mice [52]. Another tissue in which the contribution of postprandial retinyl ester uptake is thought to be important is the lung [53]. Studies in rats have shown that the retinoid content of the lung is reduced in 9-week-old rats that were raised on a retinoid-deficient diet. Neonatal retinoid supplementation of these animals was sufficient to normalize serum and liver retinoids levels, but lung levels remained low. This finding can be interpreted to indicate that the lung requires a direct contribution of postprandial retinoid to accumulate retinoid stores, presumably from chylomicrons [53].

In addition to understanding the physiological significance of postprandial retinyl ester uptake, the biochemical mechanisms involved are of interest. It was hypothesized in the mid-1990s that LpL may have a physiological role in the hydrolysis of chylomicron retinyl ester, thereby facilitating its uptake [49]. In a series of *in vitro* experiments, these authors demonstrated that LpL can catalyze the hydrolysis of retinyl ester in its four most abundant forms (retinyl palmitate, retinyl oleate, retinyl stearate, and retinyl linoleate). Triglycerides were the preferred substrate for LpL, though when the majority of triglycerides were hydrolyzed (~75%), retinyl ester hydrolysis was observed. Also, the inclusion of apolipoprotein C-II, a known LpL activator [141], further enhanced the LpL-catalyzed hydrolysis of retinyl ester. These experiments, coupled with the observation that hydrolysis of retinyl ester to retinol by LpL was associated with increased retinoid uptake by BFC-1β adipocytes, strongly suggested that LpL may have a physiological role in the extrahepatic uptake of retinoid [49]. These authors further concluded that uptake of retinol into adipocytes was not receptor mediated [49]. *In vivo* evidence in support of this hypothesis came from experiments in which the tissue levels of LpL expression were experimentally manipulated [50]. Specifically, mice over-expressing LpL in skeletal muscle show increased uptake of chylomicron retinoid into their skeletal muscle. Similarly, double-mutant mice, over-expressing human LpL in skeletal muscle, but containing a null mutation in the murine *LpL* gene, show decreased levels of uptake in the heart. In another series of experiments, LpL activity was modulated in rats by fasting. In the fasting rat, adipose tissue LpL activity is decreased, and heart and skeletal muscle activity is increased. Accordingly, fasted animals had decreased retinyl ester uptake in the adipose tissue and increased uptake in the heart and skeletal muscle, relative to control. A further dissection of LpL-facilitated uptake of chymomicron retinyl ester in the heart has recently been undertaken [142]. These authors show that uptake of chylomicron derived retinyl ester is significantly reduced in mice with a heart-specific deletion of LpL. Uptake was unaffected in CD36 knock-out mice and LpL/CD36 double knock-out mice, suggesting that this fatty-acid transporter is not essential for retinol uptake into the heart. Taken together these results indicate that, at least in skeletal muscle, heart, and adipose tissue, LpL expression and activity closely reflect the tissue’s ability to take up chylomicron retinyl ester, such that increased expression is associated with increased uptake, and *vice versa*. Interestingly, while LpL appears to facilitate chylomicron retinyl ester uptake in these tissues, other tissues which take up postprandial retinyl ester were unaffected by manipulations of LpL expression/activity, such as the kidneys and lungs. This indicates that an unidentified, LpL‑independent mechanism, exists to mediate the extra-hepatic uptake of chylomicron retinyl ester. Another interesting finding of these studies was the observation that steady-state tissue retinoid levels were unchanged. Despite increased uptake in some tissues, there was no quantitative change in tissue retinoid levels. This could suggest that the increase in retinoid uptake was not matched by an increase in the capability to store retinoid within the cell, and the excess retinoid taken up was redistributed throughout the body. This indicates that the retinoid-storage capacity of a tissue, given normal dietary retinoid intake levels, resides within the tissue. In addition to skeletal muscle, heart, and adipose tissue, LpL has also been shown to be an important mediator of chylomicron retinoid uptake in mouse and rat mammary tissue [51,52].

It is evident that extrahepatic tissues can take up postprandial retinyl ester from chylomicrons and that in RBP-deficiency this is sufficient to maintain normal retinoid homeostasis provided dietary retinoid is sufficient. The mechanisms which mediate retinyl ester uptake in the periphery are only starting to be elucidated.

### 5.3. Retinoid Homeostasis in Extrahepatic Tissues

This section will consider retinoid homeostasis and function in several specific tissues. Special attention will be given to white adipose tissue, with emphasis on the recent finding that adipose‑derived RBP may be an important signaling molecule in metabolic disease [143]. Similarly, the importance of retinoid homeostasis will be discussed in the heart, where recent studies have linked retinoid signaling and heart dysfunction [130], and the eye, where retinoid metabolism retains considerable research interest [144,145].

#### 5.3.1. White Adipose Tissue Retinoid Homeostasis

White adipose tissue is a significant site of extra-hepatic retinoid storage, accounting for ~15% of tissue retinoid stores in the adult rat [146]. Adipocytes and the stromal-vascular fraction of adipose have been shown to express key mediators of retinoid metabolism, supporting the notion that retinoid signaling is an important mediator of adipose physiology [147]. Within adult adipose tissue, RBP is predominantly expressed in adipocytes, with only weak expression in stromal-vascular cells [146]. Conversely, CRBPI was found to have relatively higher expression in the stromal-vascular cells *versus* adipocytes, though *in vitro* experiments show that retinoic acid can upregulate CRBPI expression in adipocytes [146,148]. CRBPIII has been shown to be expressed in adipose tissue homogenates [149] and in differentiated 3T3-L1 adipocytes *in vitro* [150]. However, through the use of a specific *CrbpIII*-*LacZ* mouse, expression of this gene appears to be restricted to the vascular endothelial cells of adipose tissue [151]. The mRNA transcripts for retinoic acid receptor-α, -β, and -γ have all been reported to be expressed in adipose tissue [152,153]. However, work in differentiated 3T3-L1 adipocytes reveals only a low level of RAR-α at the protein level [154]. Similarly, mRNA for the three major retinoid X receptor isoforms is also expressed in adipose/3T3-L1 adipocytes [153]. The synthesis of retinyl ester in adipose tissue was presumed to be mediated by LRAT, but this is certainly not the case, since LRAT-deficient mice have increased levels of adipose retinyl ester stores. Given the importance of LRAT in the synthesis of retinyl ester in the liver and nearly all other tissues, adipose levels of retinyl ester would be expected to be lower than WT levels, yet a greater than 3-fold increase in retinyl ester was observed [39,40,91]. This observation suggests that there is an unidentified enzyme expressed within adipocytes, the cellular site of retinoid storage within adipose tissue [146], which can synthesize retinyl ester.

There are several studies that indicate an important role for retinoid signaling in adipose tissue physiology, including the observation that retinoic acid can inhibit adipogenesis [147,155], CRBPI-deficient mice show increased adiposity [156], and CRBPIII-deficient mice have altered lipid metabolism and decreased adiposity [150]. Although CRBPIII was originally identified as a cellular retinol-binding proteins based on the ability of the recombinant protein to bind retinol, a true physiological ligand for CRBPIII has not been identified. It seems possible, even likely, that the actions of CRBPIII in lipid metabolism may involve other ligands than retinol.

#### 5.3.2. Adipose-Derived RBP and Metabolic Disease

In addition to research studying the importance of retinoid signaling on adipose tissue biology, a novel role for adipocyte-derived RBP in metabolic disease has been proposed. As discussed above, the sole established physiologic role for RBP is to mobilize hepatic retinoid stores and transport retinol in the circulation [47]. Moreover, RBP-deficient mice and RBP-deficient humans have a similar relatively mild phenotype. However, work from Kahn and colleagues shows that RBP-deficient mice display enhanced insulin sensitivity [143]. This observation, coupled with data from a number of follow-up studies, suggests that adipose-derived RBP may be a significant contributor to metabolic disease.

In 2005, Kahn and colleagues published a convincing series of studies leading to the conclusion that RBP is an important mediator of insulin resistance in obesity and type 2 diabetes [143]. It was previously known that mice with an adipose-specific deletion of the glucose transporter, GLUT4, were insulin resistant [157]. Yang *et al.* demonstrated that these mice over-express RBP in adipose tissue and that circulating levels of RBP were elevated in adipose-specific GLUT4-deficient mice. RBP expression in the liver was unchanged, suggesting that the increase in serum RBP was adipose-derived. Treatment of adipose-GLUT4-deficient mice with the PPAR-gamma agonist, rosiglitazone, was associated with improved insulin sensitivity and glucose tolerance. This improvement was paralleled by a normalization of circulating RBP levels. Interestingly, increased circulating RBP levels were found to be a common feature of multiple mouse models of insulin resistance, and serum RBP levels were also increased in human subjects with diabetes. Further evidence suggesting a role for RBP in modulating insulin sensitivity came from experimental manipulations of circulating RBP levels. Mice over-expressing human RBP in skeletal muscle have increased serum RBP levels and were found to be insulin resistant, an observation mirrored by chronic treatment with purified RBP protein. Conversely, RBP-deficient mice have enhanced insulin sensitivity, as do mice with lowered circulating RBP levels following fenretinide treatment. Taken together these studies clearly indicate that increased circulating RBP is associated with insulin resistance, whereas lowered serum RBP is associated with enhanced insulin sensitivity. The publication of these studies led to the suggestion that RBP is a novel adipokine and a potential therapeutic target in human type II diabetes [158,159,160].

Follow-up studies into the correlation between serum RBP levels and altered insulin sensitivity in humans have not resulted in consensus on this point. Several studies have confirmed the observation linking insulin resistance to high circulating levels of RBP. Indeed this concept has been expanded to suggest that the ratio of retinol to RBP more strongly correlates with insulin sensitivity [161,162,163,164,165,166]. However, other groups have been unable to show an association between high serum RBP levels and insulin resistance [167,168,169,170,171,172]. It is apparent that further study is required to definitively establish a link between elevated serum RBP levels and insulin resistance in humans. 

The studies mentioned above suggest that RBP is an important contributor to metabolic disease. What remains unclear is whether this effect is mediated through retinoid signaling or a hitherto unknown mechanism of RBP action. Studies into the molecular mechanisms of RBP-induced insulin resistance have been recently reviewed elsewhere [173,174]. There are a growing number of studies that indicate an intersection between energy balance, metabolic disease, and retinoid signaling. These developments have opened up an exciting area of retinoid biology and brought renewed attention to this essential nutrient.

#### 5.3.3. Retinoid Homeostasis in the Heart

Another expanding and important area of retinoid biology is the importance of retinoid signaling in the heart. While it has been well established that retinoic acid is an important signaling molecule during heart development in the embryo, there is a growing body of evidence that suggests this molecule is also an important factor in heart disease and cardiac remodeling [130]. In several animal models of heart disease, retinoic acid supplementation has been shown to have beneficial effects on tissue pathology. Experiments employing rat cardiomyocytes *in vitro* have shown that retinoic acid can attenuate cardiomyocyte hypertrophy induced by endothelin-1, phenylephrine, and angiotensin-II [175,176,177,178]. Following the promising results of these *in vitro* studies, experiments in whole animals have borne out the hypothesis that retinoic acid may suppress cardiac hypertrophy *in vivo*. Retinoic acid treatment has proven effective in attenuating cardiac remodeling in a rat pressure overload model (by aortic banding) and a rat model of myocardial infarction [179,180]. Experimental myocardial infarction is also associated with an increase in heart retinoid metabolism, with a significant mobilization of retinoid from the liver to the heart observed in infarcted *versus* control animals [181]. In contrast to the beneficial effects of retinoic acid in a disease setting, there is evidence to suggest that retinoid-deficiency may have adverse effects on the heart. Retinoid-deficiency in female rats has been associated with markers of cardiac remodeling and ventricular dysfunction [182]. How these animal studies relate to heart disease in humans remains largely untested. Unfortunately, in the context of acute promyelocytic leukemia, retinoic acid treatment has been associated with cardiac dysfunction in at least one case report [183]. Continuing research aimed at verifying the therapeutic potential of retinoic acid treatment in heart disease is warranted.

#### 5.3.4. Retinoid Homeostasis in the Eye

There has been a very substantial body of research carried out in the last decade aimed at defining better retinoid metabolism and actions within the eye. Notably, this literature has established many linkages between genetic eye disease and genes involved in retinoid metabolism and transport. This had long been anticipated but has only been established through groundbreaking research carried out in the last 10 to 15 years. In this review, we will only focus on a few key advances for understanding retinoid metabolism in the eye, and we will not focus on relationships of these processes with disease. A number of extensive and authoritative reviews on this topic have been published recently [145,146] and consequently the reader is referred to these reviews for more detail.

The central question regarding retinoid metabolism in vision that was left unanswered from the early work of Wald and others [184,185] concerned the mechanism through which all-*trans*-retinoid is converted to the energetically less stable 11-*cis*-retinoid that is required for vision. Based on work commencing in the mid-1980s, Rando and colleagues and others proposed the existence of an isomerohydrolase that converts all-*trans*-retinyl ester to 11-*cis*-retinol [186,187,188]. It was proposed that the isomerohydrolase utilizes energy liberated from the hydrolysis of the ester bond to drive steroisomerization from the all-*trans*- to the energetically less favorable 11-*cis*-configuration. Recently, it has been demonstrated that the abundant retinal pigment epithelium protein (RPE) RPE65 is the isomerohydrolase [189,190,191]. RPE65-deficient mice have high levels of all-*trans*-retinyl esters in their eyes and no detectable 11-*cis*-retinoids [192]. These mice are blind because they are unable to generate 11-*cis*-retinoid needed for vision. As mentioned above in Section 2.2, RPE65 structurally resembles BCMO1 and BCMO2 and, like these two other enzymes, contains a conserved Fe^2+^ binding site that is required for catalytic activity [18]. RPE65 (originally referred to as p63) had been identified in the earlier literature as a cell surface receptor for RBP, but this has proven to be incorrect [6,192].

Another important advance in understanding retinoid metabolism in the eye has been the elucidation of the mechanism through which all-*trans*-retinoid produced upon photoexcitation is removed from the photoreceptor outer segments for transport back to the RPE and reconversion to 11-*cis*-retinoid. This process has been identified to require a retina-specific ATP binding cassette transporter referred to as either ABCR or ABCA4, which localizes to the disc membranes present in rod and cone outer seqments [193,194,195]. ABCR-deficient mice show a much slower rate of removal of all-*trans*-retinal from the retina following exposure to light [196,197]. This is accompanied by an increase in retinal-phosphotidylethanolamine adducts, which have been identified as the substrates for ABCR-catalyzed removal of all-*trans*-retinal from the disc membranes to the cytoplasmic space [198,199].

Another important advancement in understanding retinoid metabolism in the eye has been the identification of retinol dehydrogenase 8 (RDH8; also referred to as photoreceptor RDH or prRDH) as one enzyme within the outer segments of the retina that catalyzes the reduction of all-*trans*-retinal to all-*trans*-retinol [200,201,202]. RDH8-deficient mice show a slower rate, by several orders of magnitude, of clearance for all-*trans*-retinal from the retina following exposure of dark adapted animals to a flash of light [200,201,202,203]. However, the kinetics of rhodopsin regeneration for RDH8-deficient mice is not different than that of WT mice, suggesting that other dehydrogenases are also involved in all-*trans*-retinal reduction to all-*trans*-retinol within the photoreceptors. Subsequent studies by Palczewski and colleagues identified RDH12 as another photoreceptor dehydrogenase that is contributes to all-*trans*-retinal reduction, although RDH12 too was observed to be dispensible [203].

In summary, the last decade has been a very exciting one for the study of retinoid metabolism in the eye. Much has been learned and the eye remains the organ where retinoid metabolism is best and most deeply understood. 
